# When less is more: regarding the use of chest X-ray instead of computed tomography in screening for pulmonary metastasis in postmolar gestational trophoblastic neoplasia

**DOI:** 10.1038/s41416-020-01209-5

**Published:** 2021-01-08

**Authors:** Antonio Braga, Kevin Meyer Elias, Neil Stuart Horowitz, Ross Stuart Berkowitz

**Affiliations:** 1 Rio de Janeiro Trophoblastic Disease Center (Maternity School of Rio de Janeiro Federal University and Antonio Pedro University Hospital of Fluminense Federal University). Postgraduate Program in Medical Sciences, Fluminense Federal University, Niterói–RJ, Brazil. Postgraduate Program in Perinatal Health, Faculty of Medicine, Rio de Janeiro, RJ Brazil; 2 New England Trophoblastic Disease Center, Division of Gynecologic Oncology, Department of Obstetrics, Gynecology and Reproductive Biology, Brigham and Women’s Hospital, Dana-Farber Cancer Institute, Harvard Medical School, Boston, MA USA

## Abstract

We discuss the use of chest computed tomography (CT) in the initial screening for gestational trophoblastic neoplasia (GTN) metastases. Chest CT does not influence overall treatment outcome or the time to remission. We endorse the FIGO recommendation for the screening of GTN metastases should be done with chest X-ray.

## Main

Since Li et al. introduced the principles of chemotherapy in the treatment of gestational trophoblastic neoplasia (GTN) with methotrexate (MTX),^1^ this disease has become highly curable, even in cases of metastases. The emergence of new antineoplastic drugs and multiagent regimens, which have made it possible to cure MTX-chemoresistant cases, has led to a dramatic fall in the lethality of this disease.^2^ Consequently, it has become vitally important to identify which patients are unlikely to respond to single-agent chemotherapy and require upfront multiagent therapy.

Since 2000, specialists devoted to the treatment of GTN have used the staging and prognostic scoring system developed by the World Health Organization (WHO) and the International Federation of Gynecology and Obstetrics (FIGO) to determine which patients are at low-risk to develop chemoresistance and therefore should be treated with single-agent chemotherapy (score ≤ 6), and which patients are at high-risk to be resistant to single-agent chemotherapy and require multiagent treatment (score ≥ 7) (Table 1).^3^

**Table 1:** WHO/FIGO staging and classification of gestational trophoblastic disease^a^.

GTN: FIGO staging and classification (Washington, 2000)				
FIGO anatomic staging:				
Stage I: Disease confined to the uterus				
Stage II: GTN extends outside of the uterus, but is limited to the genital structures (adnexa, vagina, broad ligament)				
Stage III: GTN extends to the lungs, with or without known genital tract involvement				
Stage IV: All other metastatic sites				

Modified WHO prognostic scoring system as adapted by FIGO				
Prognostic factors	Score			
	0	1	2	4
Age (years)	<40	≥40	–	–
Antecedent gestation	Mole	Abortion	Term	–
Interval^b^ (months)	<4	4–6	7–12	>12
Pretreatment serum hCG (IU/l)	<10^3^	10^3^ to <10^4^	10^4^ to <10^5^	>10^5^
Largest tumour size (including uterus)	<3	3 to 4	≥5	–
Site of metastases	Lung	Spleen, kidney	Gastrointestinal tract	Brain, liver
Number of metastases	–	1–4	5–8	>8
Previous failed chemotherapy	–	–	Single drug	Two or more drugs

*WHO/FIGO* World Health Organization/International Federation of Gynecology and Obstetrics, *GTN* Gestational trophoblastic neoplasia, *hCG* Human chorionic gonadotropin.

^a^This Table has been reproduced from FIGO Oncology Committee (2002), FIGO staging for gestational trophoblastic neoplasia 2000. Int. J. Gynaecol. Obstet. **77**, 285–287 with permission. ©  John Wiley and Sons (Copyright License Number 4945060393283).

^b^Interval (in months) between the end of antecedent gestation (when known) and the beginning of chemotherapy.

The use of this classification has become the cornerstone of GTN treatment. The prognostic factors involved in this classification are age, index gestation, interval between the end of antecedent gestation and the beginning of chemotherapy, pretreatment serum human chorionic gonadotropin (hCG) level, largest tumour size (including uterus), site and number of metastases and previous failed chemotherapy. As the most common site of GTN metastasis is the  lung,^4^ the size and number of lung metastases is fundamental for a correct assessment of the WHO/FIGO prognostic score. Although FIGO expressly recommends screening for GTN lung metastases using chest X-ray,^3,5^ the use of chest computed tomography (CT) has become increasingly common in cancer staging not only because of its higher sensitivity for detecting metastatic nodules, but also for more accurate measurement of tumour size.

In this issue of *British Journal of Cancer*, Parker et al., from Sheffield Trophoblastic Disease Reference Centre, conclude chest CT improves prediction of single-agent chemotherapy resistance, but does not influence overall treatment outcome or the time to hCG normalisation.^6^ The authors report that chest CT increased the total number of metastases detected when compared with chest X-ray, increasing the FIGO score in 188 of 589 patients (31.9%) and even reclassifying 43 (7.3%) patients from the low- to high-risk group. Although the chest CT better identified patients who would experience resistance to primary single-agent chemotherapy, all of these patients ultimately achieved remission with other regimens. When patients are categorised as having high-risk GTN, they are generally treated with more toxic multiagent chemotherapy.^3–5^ Among the 43 patients who were reclassified as having high-risk GTN, 23 (53.5%) were cured with single-agent chemotherapy. Probably one-half of the patients who were reclassified as high-risk could have been successfully treated with less toxic single-agent chemotherapy and could have been spared multiagent chemotherapy if the chest CT finding was not employed in the prognostic scoring system.

The article by Parker et al. clearly shows that chest CT should not replace chest X-ray in the management of GTN.^6^ It is vitally important that the findings on chest CT not be used in assigning WHO/FIGO risk score.^3,5^ If chest CT findings are used to determine risk score, patients with low-risk non-metastatic or metastatic disease may be categorised as having high-risk metastatic disease and be unnecessarily treated with more toxic combination chemotherapy. The primary goal in the treatment of GTN is to achieve the highest remission rate with the lowest possible morbidity—or as Hippocrates expressed: *primum non nocere* (first, do no harm!). The use of chest CT to assign a prognostic risk score is in conflict with the best goals of treatment, this is without even taking into account the extra radiation exposure and the costs involved in performing the CT. There are several examples in medicine where technological advances have not always improved the patient’s care. Parker et al.^6^ support the FIGO recommendation for the screening of GTN metastases should be done with chest X-ray. It is worth remembering: less, sometimes, is more!

## Data Availability

Not applicable.
